# Proteomic and Clinical Characterization of Active vs. Quiescent Pterygium With Elevated Thrombospondin-1

**DOI:** 10.1167/tvst.15.6.30

**Published:** 2026-06-24

**Authors:** Jiaxin Han, Qianwen Gong, Kexin Li, Huijuan Chen, Jian Zhao, Yixuan Tong, Lingying Ye, Bintao Xie, Xiaolu You, Fan Lu, Liang Hu

**Affiliations:** 1 National Clinical Research Center for Ocular Diseases, Eye Hospital, Wenzhou Medical University, Wenzhou, China; 2 State Key Laboratory of Ophthalmology, Optometry and Visual Science, Eye Hospital, Wenzhou Medical University, Wenzhou, China

**Keywords:** pterygium, anterior segment-optical coherence tomography angiography, biomarker, proteomics, THBS-1

## Abstract

**Purpose:**

To investigate proteomic and clinical changes in active- and quiescent-stage pterygium and identify biomarkers.

**Methods:**

This study included 15 and 14 patients with quiescent- and active-stage pterygium, respectively. Clinical parameters (length, area, thickness, vessel density, and hemodynamics) were assessed using slit-lamp, anterior segment-optical coherence tomography angiography, and functional slit-lamp biomicroscopy. Label-free proteomics was performed on excised tissues. Thrombospondin-1 (THBS1) was validated via enzyme-linked immunosorbent assay, immunohistochemistry, and reverse transcription quantitative polymerase chain reaction. Correlation and receiver operating characteristic analyses were also conducted.

**Results:**

Compared with the quiescent stage, active pterygium exhibited significantly greater corneal invasion length, area, thickness, and vessel density, along with reduced vessel length. Proteomic analysis revealed 7 upregulated differentially expressed proteins whose pathways were mainly immune related and 15 downregulated differentially expressed proteins. THBS1 was significantly elevated in active tissue, confirmed by enzyme-linked immunosorbent assay, strong immunohistochemical staining, and higher messenger RNA levels. Receiver operating characteristic analysis supported THBS1 as a potential biomarker, yielding an area under the curve of 0.81 for distinguishing active-stage pterygium.

**Conclusions:**

Immune dysregulation and neovascularization may play an important role in pterygium progression. THBS1 may serve as a potential biomarker for predicting pterygium development and monitoring its progression.

**Translational Relevance:**

This work translates the discovery of elevated thrombospondin-1 into a potential clinical biomarker, bridging proteomic findings to the assessment of pterygium activity.

## Introduction

Pterygium, a common ocular surface disease, presents as a fibrovascular growth on the bulbar conjunctiva that invades the cornea. Pterygium can range from small, atrophic quiescent lesions to large, aggressive, rapidly growing fibrovascular lesions.^1^ Depending on its progression, pterygium can be divided into active and quiescent stages.^2^ The active stage progresses more rapidly than the quiescent-stage pterygium. Surgery is the mainstay of treatment for this disease, but recurrence rates still range from 2% to 80%.^3^^–^^5^ Studies have found that active pterygium is more likely to recur postoperatively.^6^^,^^7^

Anterior segment-optical coherence tomography angiography (AS-OCTA) is a noninvasive and noncontact device that allows the visualization of blood vessels by continuous B-scan imaging of red blood cell motility in blood vessels^8^; it is easily reproducible and reliable.^9^ Zhao et al.^10^ studied pterygium using AS-OCTA and found that the vascular density was higher than that of normal conjunctiva. However, the changes in vascular density on AS-OCTA and the hemodynamics of the different stages of pterygium remain unknown.

Proteomics technologies that have emerged in recent years can characterize and quantify proteins in high throughput and large scale with high sensitivity, providing new technical routes and research ideas for the study of proteins of interest. One study^11^ used proteomics to investigate the differences between pterygium and normal conjunctiva, revealing the involvement of inflammation and defense responses in the development of pterygium. However, the precise molecular mechanisms underlying the divergent behavior of pterygium in different states remain unclear.

Herein, we used a label-free proteomics approach to study the protein composition of different stages of pterygium. Additionally, by correlating the proteome of pterygium with clinical features, we sought to gain new insights into the mechanisms underlying pterygium development and identify potential biomarkers for active pterygium.

## Methods

### Patients and Samples

A total of 29 patients were prospectively enrolled between July 2022 and March 2023 from the Eye Hospital of Wenzhou Medical University. Inclusion criteria were (1) the presence of primary nasal pterygium; (2) no prior pterygium surgery; and (3) a surgical indication. Patients with a history of ocular surgery, recurrent pterygium, ocular trauma, contact lens wear, conjunctivitis, corneal scarring, glaucoma, use of medications that alter the appearance of blood vessels, and systemic diseases, such as diabetes, were excluded. All patients received topical levofloxacin eye drops for 3 days before surgery as routine preoperative prophylaxis, and no other ocular medications were used before surgery. This study was approved by the Research Ethics Committee of Wenzhou Medical University (code: 2022-126-K-96-01) and adhered to the tenets of the Declaration of Helsinki. Participants received a full explanation of the procedures and provided informed consent for participation before inclusion in the study. All pterygium excisions with conjunctival autotransplantation were performed by the same surgeon. Nasal pterygiums tissues were stored at −80°C until processing.

### Clinical Assessment

#### Pterygium Grouping

To objectively define pterygium activity, we developed the Pterygium Activity Index (PAI), which quantifies the classic clinical features of disease stage. The PAI was calculated by combining the grading scales proposed by Tan^12^ for tissue transparency (1–3) and by Kim et al.^13^ for vascularity (1–3), reflecting the core pathological changes of hypertrophy/congestion vs. thin, noncongested tissue.^14^ Two masked ophthalmologists independently assigned these grades from slit-lamp photographs; a third senior ophthalmologist resolved any disagreements. The final PAI (range, 2–6) was calculated as the sum of both scores. We classified pterygia with a PAI of ≤3 as quiescent stage and those with a PAI of ≥4 as active stage.

### Slit-Lamp Assessment

All patients underwent anterior segment photography using a slit-lamp (SLE-8E, KangHua RuiMing, Chongqing, China). Quantification of the area of the pterygium head was performed with Image J using the fiber grading method.^15^ The length of pterygium, defined as the distance from the limbus to the edge of pterygium, was also recorded.^16^^,^^17^

### AS-OCTA Assessment

All scans were performed by the same physician using a swept-source OCTA (VG100D, SVision Imaging, Luoyang, China), which operates at approximately 1050 nm, with a scan speed of 100,000 A-scans per second and a wide field of 56°. Images were acquired in AS Angio scan mode with a scan depth of 4.1 mm. Each 6 × 6 mm^2^ volume scan comprised 384 A-scans per B-scan with a total of 384 B-scan locations. OCTA B-scans were examined, and manual correction was performed when segmentation errors were detected. Patients looked at a fixation point located on the temporal side of the eye, and the scanning area was a 6 × 6 mm area tangent to the nasal side of the corneal limbus. Pterygium vessel density was defined as the percentage of vessel area within the scanned region and was measured automatically using the built-in software (Version 1.44.2; SVision Imaging, Luoyang, China). Pterygium thickness was measured manually at the limbus, with the horizontal axis aligned with the long axis of the pterygium, using the caliper function of the built-in software.^17^ Both pterygium vessel density and thickness were measured three times and the average value was used for analysis.^18^

### Functional Slit-Lamp Biomicroscope Assessment

The functional slit-lamp biomicroscope imaging system and imaging protocol have been described in our previous studies.^17^ Six different locations, approximately 1 mm away from the limbus on the nasal bulbar conjunctiva, were imaged for the measurements of axial blood flow velocity (mm/s), cross-sectional blood flow velocity (mm/s), vessel diameter (µm), vessel length (µm), and flow volume (pL/s).

### Proteomic Profiling and Candidate Protein Validation

#### Label‑Free Quantitative Proteomics

Proteins were extracted from nasal pterygium tissue and digested using the Thermo EasyPep Mini MS Sample Prep Kit (Thermo Fisher Scientific, Waltham, MA; cat# A4006). Peptides were desalted, reconstituted in 0.1% formic acid, and quantified. Liquid chromatography-tandem mass spectrometry analysis was performed on an EASY‑nLC 1200 system coupled to an Orbitrap Fusion Lumos Tribrid mass spectrometer (Thermo Fisher Scientific). Peptides were separated on a 50‑cm EASY‑Spray C18 column using a 67‑minute gradient of 3% to 100% acetonitrile (0.1% formic acid) at 300 nL/min. Full mass spectrometry scans were acquired at 60,000 resolution (m/z 350–1500), followed by data‑dependent tandem mass spectrometry scans at 15,000 resolution.

#### Enzyme-Linked Immunosorbent Assay (ELISA)

Thirteen pterygium tissue samples (seven quiescent‑stage and six active‑stage) were used to quantify thrombospondin-1 (THBS1) protein levels by ELISA. Frozen tissues stored at −80 °C were homogenized in phosphate‑buffered saline. After centrifugation, total protein concentration in the supernatant was determined using a bicinchoninic acid assay kit (Beyotime Biotechnology, Shanghai, China; Cat. #P0009). Samples were then analyzed with a human THBS1 ELISA kit (Wuhan Mosak Biotechnology Co., Hubei, China; Cat. #KT99027‑96T) according to the manufacturer's instructions. Absorbance was measured at 450 nm.

#### Immunohistochemical Staining

Pterygium tissue samples were fixed in 4% paraformaldehyde, dehydrated, embedded in paraffin, and sectioned at a thickness of 5 µm. For immunohistochemical (IHC) staining, sections were deparaffinized, rehydrated, and subjected to heat-induced antigen retrieval in sodium citrate buffer (pH 6.0; Maixin Biotech, Fuzhou, China; Cat. #MVS-0100). Endogenous peroxidase activity was quenched by incubation with 3% hydrogen peroxide. Sections were incubated overnight at 4°C with a primary antibody against THBS1 (1:100 dilution; Maiwei (Shanghai) Biotechnology Co., Shanghai, China; Cat. #P105217). Following washes, immunodetection was performed using the Elivision plus Polyer horseradish peroxidase IHC Kit (Maiwei (Shanghai) Biotechnology Co., Cat. #KIT-9901) with DAB chromogen (Maiwei (Shanghai) Biotechnology Co., Cat. #DAB-0031), strictly according to the manufacturer's instructions.

IHC staining for THBS1 was quantified using a semiquantitative H‑score method. The expression of THBS1 was quantified on five randomly selected high‑power fields (400× magnification) per section using the IHC Profiler plugin in ImageJ.^19^ For each field, an H‑score was calculated as: H‑score = 3 × (% high positive) + 2 × (% positive) + 1 × (% low positive).^20^ The percentage of negative-stained areas was included in the plugin's analysis but assigned a weight of zero in this scoring system.

#### Reverse Transcription Quantitative Polymerase Chain Reaction (RT‑qPCR)

Total RNA was extracted from tissue samples using the FastPure Cell/Tissue Total RNA Isolation Kit (Vazyme Biotech, Nanjing, China; Cat. #RC112-01) according to the manufacturer's instructions. RNA was reverse transcribed into complementary DNA using the HiScript III RT SuperMix kit (Vazyme Biotech, Cat. #R323-01). RT-qPCR was performed on complementary DNA using ChamQ Universal SYBR qPCR Master Mix (Vazyme Biotech, Cat. # Q711-03) on an ABI Q6 Real-Time PCR System (Life Technologies, Grand Island, NY). The primers used for THBS1 were: forward, 5′‑GCCACAGTTCCTGATGGAG‑3′ and reverse, 5′‑CCATGGAGACCAGCCATC‑3′. The relative transcription was analyzed using the 2^−ΔΔCt^ method with β-actin as an internal reference gene.

### Statistical and Bioinformatics Analyses

Clinical data were analyzed using SPSS (version 26.0 for Windows; IBM, Armonk, NY). Sex differences were analyzed with the χ^2^ test. The Shapiro–Wilk test was used to assess data normality. Differences in the clinical features were compared using the *t*-test or nonparametric tests according to the data normality. All continuous data were presented as mean ± standard deviation. For correlation analysis between two continuous, normally distributed variables, Pearson's correlation coefficient was calculated. Statistical significance was set at a *P* value of <0.05. Receiver operating characteristic (ROC) analysis was used to examine the classification accuracy of upregulated proteins and key clinical parameters. The area under the curve (AUC) value with 95% confidence intervals (95% CIs) are reported.

For differential expression analysis, proteins detected in ≥70% of samples within each group were retained for statistical comparison. Proteins meeting this criterion were considered reliably quantified and included in downstream differential expression analysis. For proteomic data, the Student *t*-test was used to evaluate significant differences. For initial screening, candidate differentially expressed proteins (DEPs) were defined using a threshold of |fold change| of >1.5 and a *P* value of <0.05. We acknowledge that this approach prioritizes sensitivity for discovery. Heatmaps were generated using the Complexheatmap R package (R Version 4.2.1).^21^ Protein Gene Ontology (GO) database analysis and Kyoto Encyclopedia of Genes and Genomes (KEGG) pathway enrichment analysis were performed to examine the biological pathways of the DEPs.^22^ Furthermore, protein–protein interaction (PPI) analysis was conducted and visualized using the String database (http://string-db.org/).

To address the limitations of single-protein analysis and to test for coordinated biological changes at a systems level, a Gene Set Enrichment Analysis (GSEA) was performed using the full ranked list of all quantified proteins. Two curated gene set collections were used: the GO Biological Processes and the MSigDB Hallmark gene sets. In accordance with standard practice for discovery-phase analyses, gene sets with a false discovery rate of <0.25 were considered statistically significant.^23^^,^^24^

## Results

### Clinical Assessment

**Figure 1.**
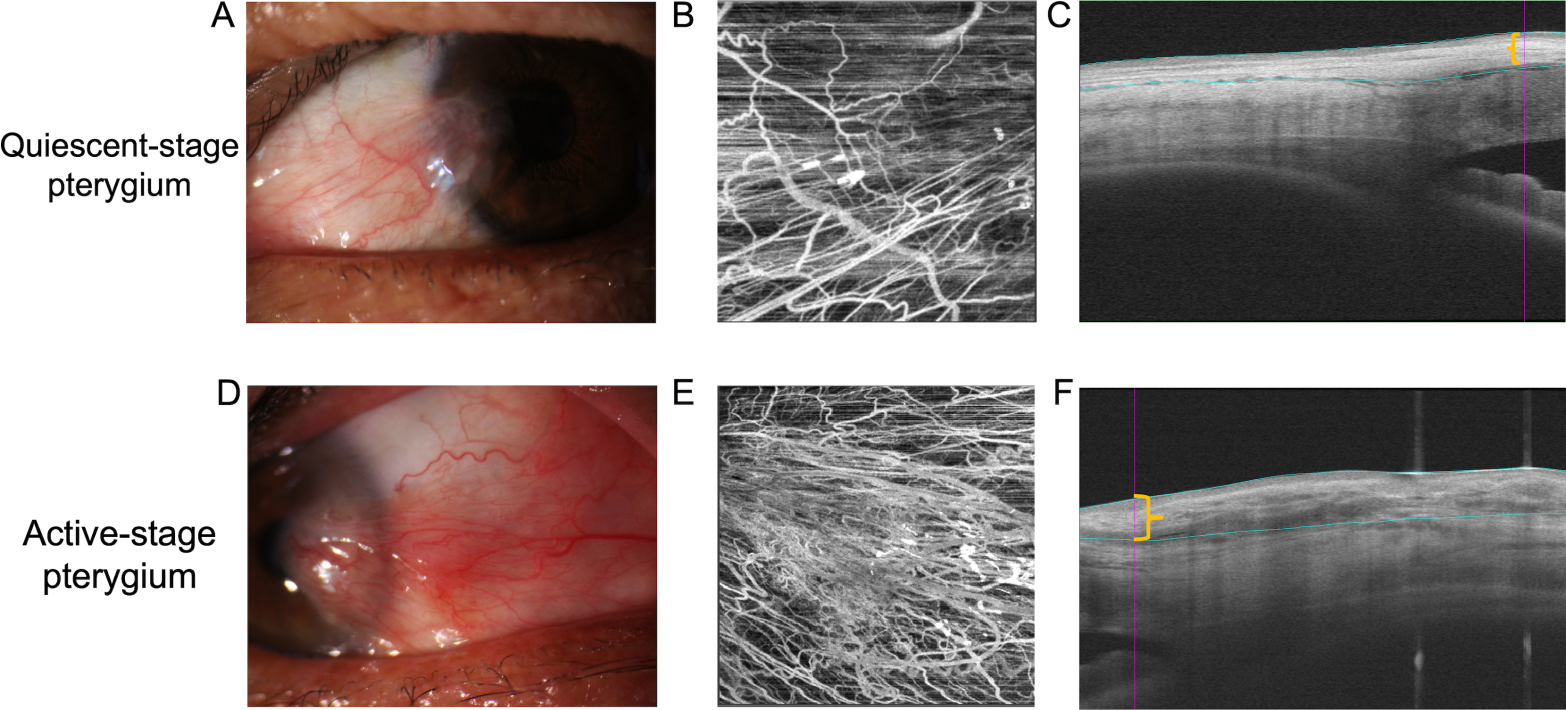
Representative images of active- and quiescent-stage pterygium. (**A**, **D**) Diffuse illumination images obtained by slit-lamp photography. Pterygium appears more congested and hypertrophic in the active stage than in the quiescent stage. (**B**, **E**) En face anterior segment optical coherence tomography angiography images showing blood flow signals. Vessel density is higher in active-stage pterygium than in quiescent-stage pterygium. (**C**, **F**) B-scans showing pterygium thickness. The *blue line* indicates the segmentation of the pterygium, the *red line* indicates the corneal margin, and the *yellow brackets* indicate the measurement site for pterygium thickness.

Age, sex, and disease duration were comparable between the quiescent-stage and active-stage groups (all *P* > 0.05). Pterygium length, area, and thickness were significantly greater in the active-stage group than in the quiescent-stage group (Table). Vessel density was also significantly higher in the active-stage group, whereas vessel length was significantly lower. No significant differences were observed in vessel diameter, axial blood flow velocity, cross-sectional blood flow velocity, or flow volume between the two groups (*P* > 0.05) (Table; Fig. 1).

**Table.** Clinical Parameters of Quiescent-Stage and Active-Stage Pterygium

	Quiescent-Stage Pterygium	Active-Stage Pterygium	*P* Value	Test Statistic
No. of subjects	15	14		
Sex (male/female)	6/9	8/6	0.466	
Disease duration (years)	7.27 ± 3.63	7.93 ± 4.51	0.880	−0.159
Age (years)	59.20 ± 10.26	65.21 ± 12.27	0.163	−1.436
Length of pterygium (mm)	3.27 ± 0.85	4.26 ± 1.01	0.008^*^	−2.847
Area of pterygium (mm^2^)	13.62 ± 7.73	18.46 ± 7.69	0.036^†^	−2.095
Thickness of pterygium (µm)	360.45 ± 104.84	472.44 ± 123.59	0.014^*^	−2.638
Vessel density (%)	63.66 ± 5.74	68.86 ± 4.64	0.013^*^	−2.671
Vessel diameter (µm)	16.89 ± 2.18	16.50 ± 2.17	0.629	0.489
Vessel length (µm)	158.04 ± 24.64	138.56 ± 17.63	0.022^*^	2.432
Va (mm/s)	0.57 ± 0.22	0.63 ± 0.32	0.948	−0.065
Vs (mm/s)	0.41 ± 0.16	0.45 ± 0.22	0.711	−0.371
Flow volume (pL/s)	119.17 ± 57.16	127.87 ± 95.94	0.477	−0.72

Va, axial blood flow velocity; Vs, cross-sectional blood flow velocity.

Data are expressed as mean ± standard deviation.

* *P* < 0.05 (*t*-test).

† *P* < 0.05 (Mann–Whitney *U* test).

### Label-Free Proteomics

#### Identification of Expressed Proteins

**Figure 2.**
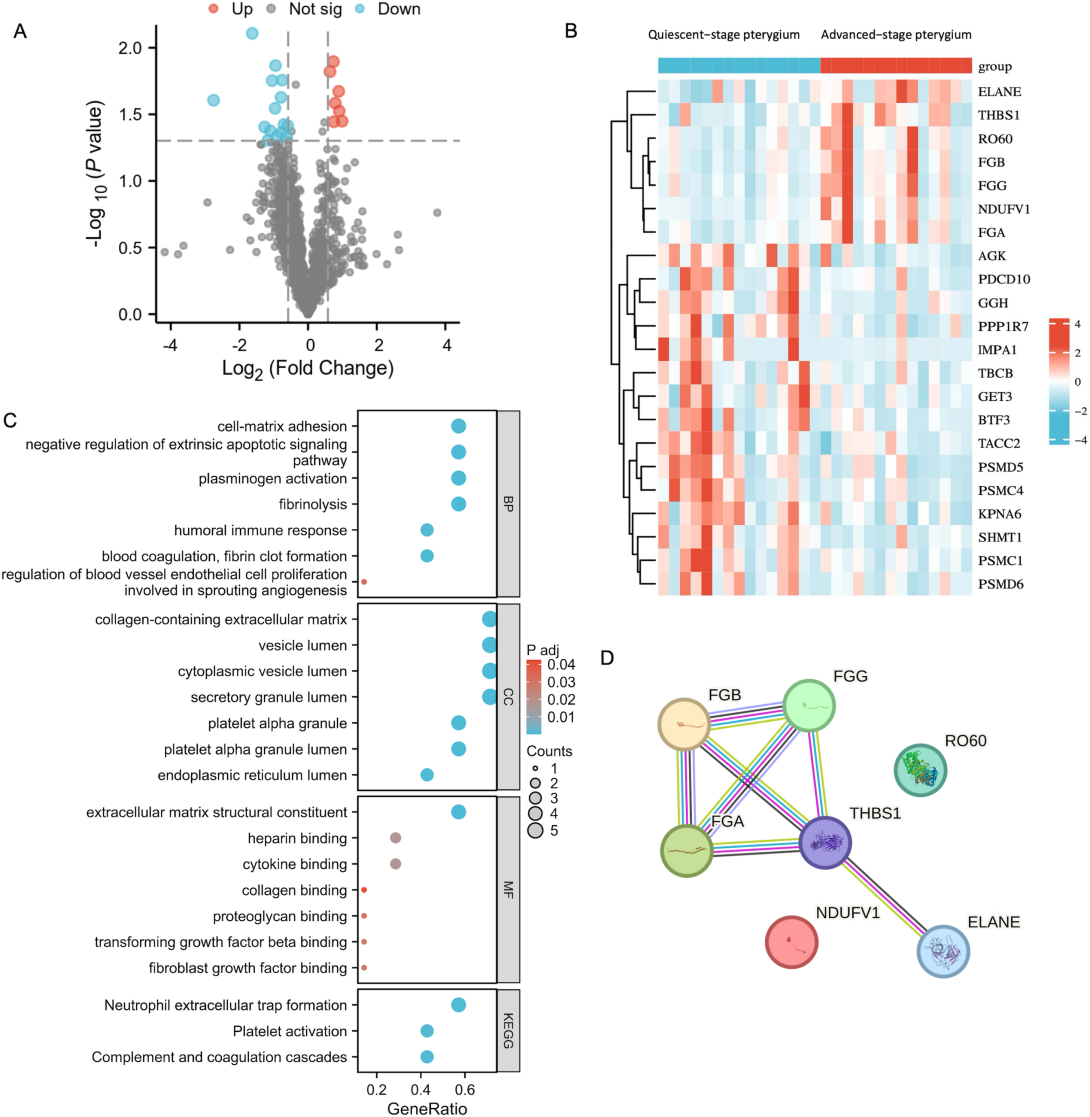
Differential proteomic profiling and functional enrichment analysis. (**A**) Volcano plot of differentially expressed proteins (DEPs). (**B**) Heatmap showing the hierarchical clustering of samples and DEPs. The expression of DEPs in different samples is shown in *red* (high expression) and *blue* (low expression). (**C**) Gene Ontology (GO) and Kyoto Encyclopedia of Genes and Genomes (KEGG) enrichment analyses of upregulated DEPs. Biological process (BP), cellular component (CC), molecular function (MF), and KEGG pathway categories are displayed. *Dot size* represents gene count, and *color intensity* corresponds with the adjusted *P* value. (**D**) Protein–protein interaction network of upregulated proteins.

Label-free proteomic profiling of 29 samples identified a total of 4721 proteins. Proteins detected in ≥70% of samples within each group were retained, resulting in 2713 high-confidence proteins for subsequent differential expression analysis. Compared with quiescent-stage pterygium, active-stage pterygium exhibited 7 upregulated and 15 downregulated DEPs (Fig. 2A) (Supplementary Tables S1 and S2). Hierarchical clustering of DEPs demonstrated a clear separation between active- and quiescent-stage pterygium (Fig. 2B).

#### Protein Function Classification and Enrichment Analysis

**Figure 3.**
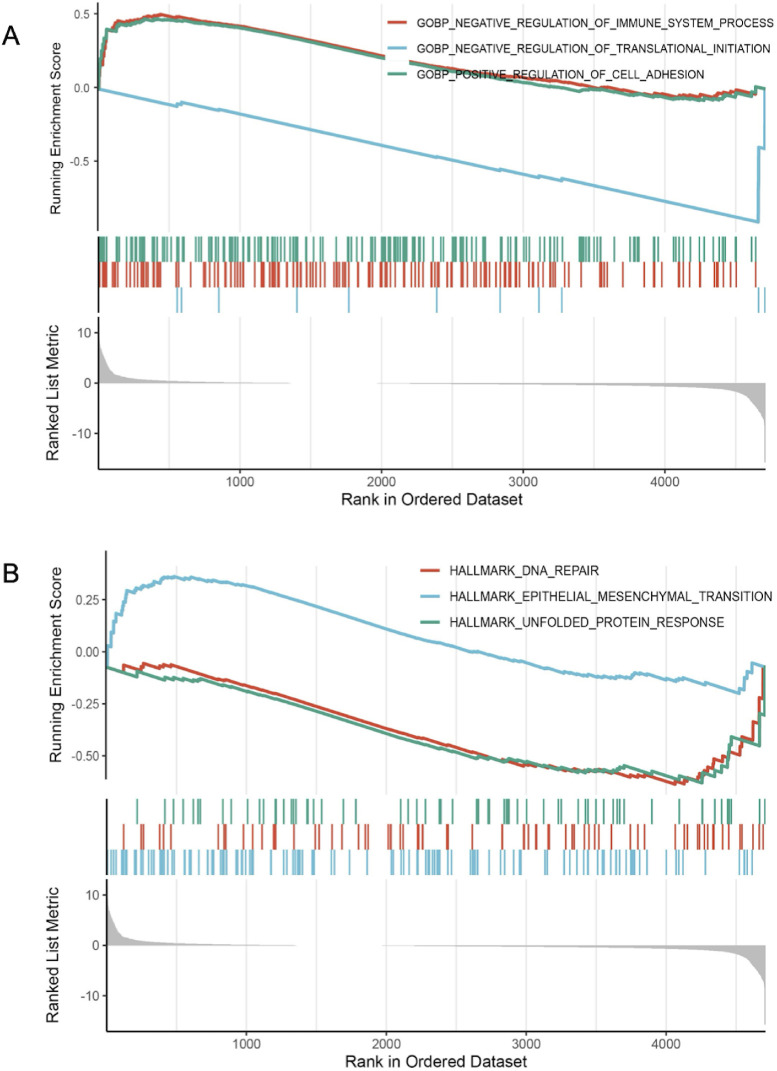
Gene set enrichment analysis (GSEA). (**A**) GSEA of GO biological processes. (**B**) GSEA of Hallmark gene sets.

DEPs in active- and quiescent-stage pterygium were associated with different biological processes, cellular components, and molecular functions (Fig. 3C). For upregulated DEPs, biological process enrichment primarily included hemostasis-related terms (fibrinolysis, plasminogen activation, and blood coagulation), cell-matrix adhesion, sprouting angiogenesis, and negative regulation of extrinsic apoptotic signaling, largely driven by THBS1, FGA, FGB, and FGG. Additionally, the response to ultraviolet radiation, a key environmental risk factor for pterygium,^25^ was notably upregulated, involving proteins such as RO60 and ELANE. At the cellular component level, upregulated proteins were enriched in luminal structures (platelet alpha granule lumen, secretory granule lumen, cytoplasmic vesicle lumen, and vesicle lumen) and the collagen-containing extracellular matrix; all of these cellular components are associated with THBS1. Molecular function analysis revealed elevated binding activities for fibroblast growth factor, transforming growth factor-β, and collagen—all of which align with the known functions of THBS1. KEGG pathway analysis highlighted enrichment in neutrophil extracellular trap (NET) formation, complement and coagulation cascades, and platelet activation, pathways that are primarily related to immune function. In contrast, downregulated DEPs were chiefly involved in proteasome-mediated protein degradation and metabolic processes, with cellular components predominantly localized to proteasome complexes and molecular functions related to enzyme catalytic activities.

#### PPI Network

PPI network analysis elucidated functional interactions among the upregulated DEPs (Fig. 2D). The network revealed direct interactions among five of the eight upregulated proteins (FGA, FGB, FGG, THBS1, and ELANE), which are functionally associated with the complement and coagulation cascades. Among these, THBS1 exhibited the highest number of interaction partners (degree), positioning it as a central hub within the network.

#### GSEA

GSEA performed on the global proteomic profile revealed coordinated alterations in biological functions during pterygium progression. GO Biological Processes identified several pathways that were significantly enriched at the standard discovery threshold. These significant processes highlighted two convergent themes. First, terms related to immune modulation, such as negative regulation of immune system process (normalized enrichment score [NES] = 1.63) and negative regulation of adaptive immune response (NES = 1.84), were enriched, suggesting an actively regulated or suppressive immune microenvironment in advanced pterygium. Second, processes governing tissue structure and cellular interaction, including positive regulation of cell adhesion (NES = 1.56) and external encapsulating structure organization (NES = 1.65), were also prominent (Fig. 3A). Concomitantly, several processes involved in fundamental cellular homeostasis, such as the regulation of transcriptional/translational elongation and sulfur amino acid metabolism, exhibited a coordinated downregulation (Supplementary Table S3).

Analysis of MSigDB Hallmark gene sets further identified biologically pertinent, albeit nominally significant, coordinated changes: the epithelial–mesenchymal transition signature was positively enriched, whereas the DNA repair pathway was negatively enriched, suggesting a concomitant activation of tissue-remodeling programs and a potential decline in genomic maintenance (Fig. 3B). Collectively, these results indicate that pterygium progression is underpinned by a systematic dysregulation of key biological processes governing immune responses and tissue structure, alongside perturbations in higher-order pathway signatures related to cellular plasticity and DNA damage response.

### Correlation Between DEPs and Clinical Parameters

**Figure 4.**
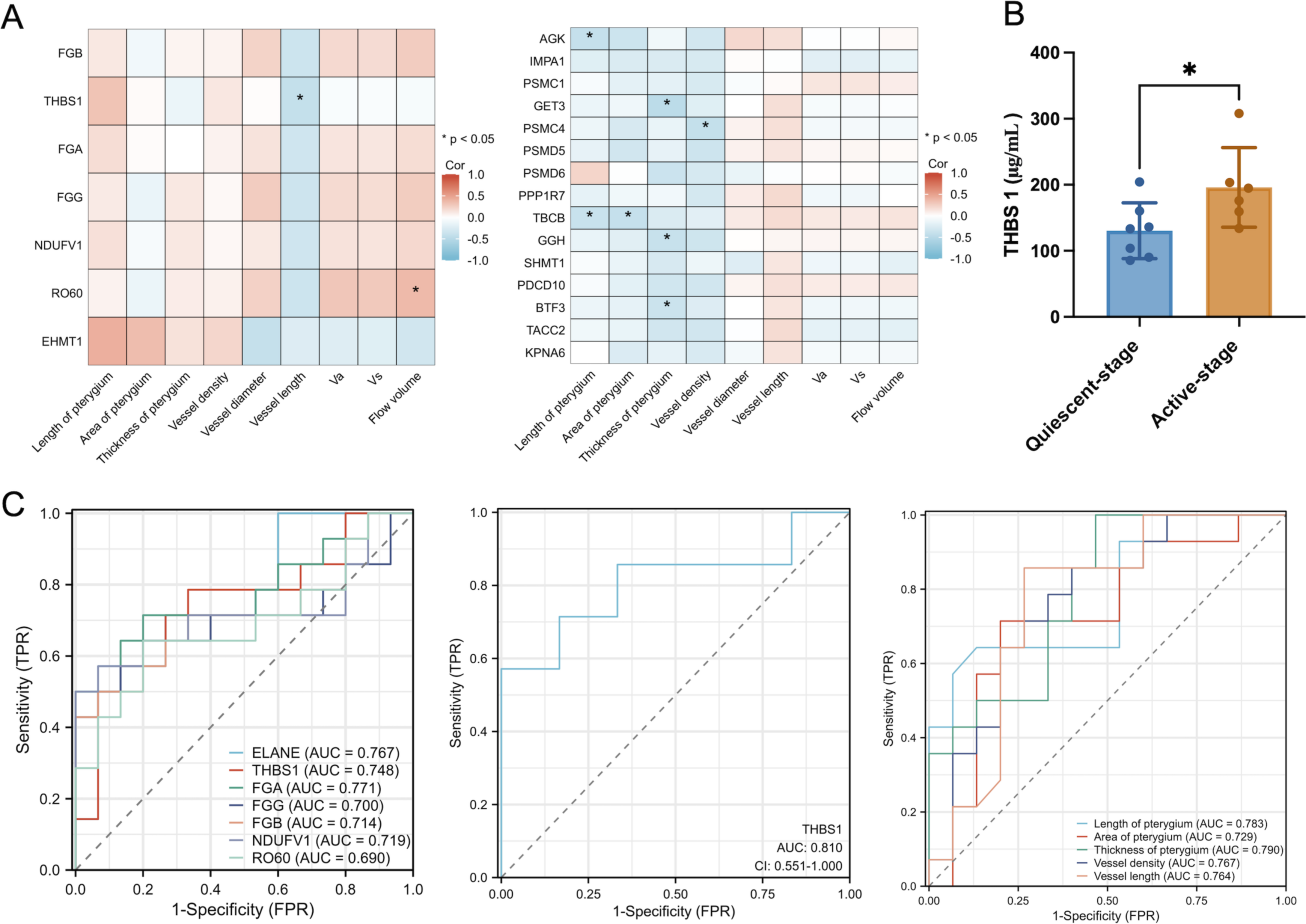
Clinical correlations, validation, and diagnostic performance of differentially expressed proteins (DEPs). (**A**) Heatmap showing correlations between DEPs and clinical parameters. *P < 0.05. (**B**) Validation of thrombospondin-1 (THBS1) expression by enzyme-linked immunosorbent assay (ELISA). *P < 0.05 (*t*-test). (**C**) Receiver operating characteristic (ROC) curve analyses. (*Left*) ROC curves of upregulated DEPs for distinguishing active-stage from quiescent-stage pterygium based on proteomic data. (*Middle*) ROC curve of THBS1 based on ELISA measurements. (*Right*) ROC curves of significantly different clinical parameters.

We examined correlations between the 22 DEPs and clinical parameters, including pterygium length, area, thickness, vessel density, axial and cross-sectional blood flow velocity, vessel diameter, vessel length, and flow volume (Fig. 4A). Significant associations (*P* < 0.05) were identified for eight proteins. Among the upregulated DEPs, THBS1 was negatively correlated with vessel length (*r* = −0.418; *P* = 0.024), whereas RO60 showed a weak positive correlation with flow volume (*r* = 0.371; *P* = 0.047). Downregulated DEPs were primarily negatively correlated with pterygium length, area, thickness, and vessel density. These correlations help to elucidate the relationship between protein expression and clinical features, supporting the potential biomarker role of these DEPs.

### ELISA Validation of THBS1

Given its central role in the PPI network and correlation with clinical parameters, THBS1 was selected for further validation. ELISA revealed that THBS1 concentration was significantly higher in active-stage pterygium (195.92 ± 60.24 µg/mL) than in quiescent-stage pterygium (130.50 ± 42.29 µg/mL; P = 0.042) (Fig. 4B).

### ROC Analysis

To evaluate the biomarker potential of protein levels for active-stage pterygium, ROC analysis was performed. Based on the proteomic dataset, all upregulated DEPs except RO60 and FGG showed significant discriminative capacity (AUC > 0.7) (Fig. 4C, left; Supplementary Table S4). THBS1, prioritized for orthogonal validation, achieved an AUC of 0.748 (95% CI, 0.56–0.93; *P* = 0.009) in the discovery phase. Independent validation using ELISA-measured THBS1 yielded a consistent AUC of 0.81 (95% CI, 0.56–1.06; *P* = 0.015) (Fig. 4C, middle). The optimal cutoff (133.70 µg/mL) maximized Youden's index, providing 100% sensitivity and 57.1% specificity. These results confirm the robust potential of THBS1 as a biomarker for distinguishing active-stage pterygium. ROC analysis of clinical parameters that differed significantly between groups showed AUCs ranging from 0.7 to 0.8 (Fig. 4C, right; Supplementary Table S4).

### IHC Analysis of THBS1

**Figure 5.**
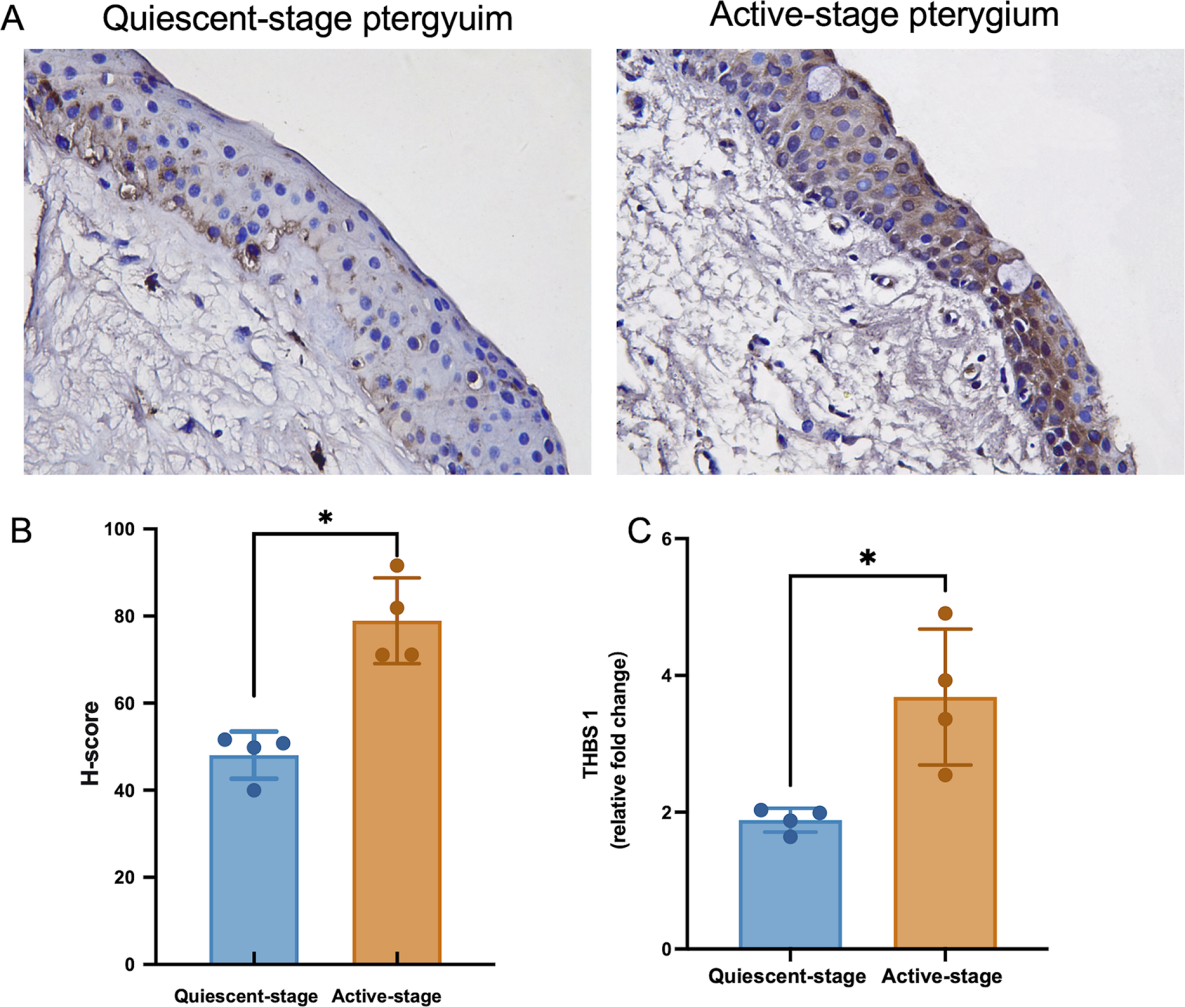
Validation of thrombospondin-1 (THBS1) expression in pterygium tissues. (**A**) Representative images (400× magnification) of THBS1 immunostaining (*brown*) with hematoxylin counterstain (*blue*) in quiescent-stage (*left*) and active-stage (*right*) pterygium. (**B**) Quantitative analysis of THBS1 protein expression using H‑score based on IHC staining. *P = 0.029 (Mann–Whitney *U* test), *n* = 4. (**C**) Messenger RNA expression level of THBS1 determined by reverse transcription quantitative polymerase chain reaction. *P = 0.012 (*t*-test), *n* = 4.

IHC analysis localized and semiquantified THBS1 protein expression. Stronger THBS1 immunostaining was observed in active-stage compared with quiescent-stage pterygium tissues. Quantitatively, the H-score was significantly higher in the active group (78.92 ± 4.92) than in the quiescent group (48.07 ± 2.71; Mann–Whitney U = 16.000; *P* = 0.029), providing independent histological validation of THBS1 upregulation (Figs. 5A, 5B).

### THBS1 Messenger RNA Expression Analysis

RT‑qPCR analysis demonstrated that THBS1 messenger RNA level was significantly higher in active-stage pterygium (3.68 ± 0.99) than in quiescent-stage pterygium (1.88 ± 0.17; *t* = −3.571; *P* = 0.012), confirming upregulation at the transcriptional level consistent with protein findings (Fig. 5C).

## Discussion

This study indicates that immunity and angiogenesis play an important role in the development of pterygium, and THBS1 has potential as a marker for active-stage pterygium.

Clinically, effectively controlling disease progression remains challenging. Furthermore, a more active pterygium is associated with a higher risk of postoperative recurrence, making recurrence reduction an urgent issue. Therefore, it is necessary to explore differences between different types of pterygia and to unveil their underlying developmental mechanisms. In our study, active pterygium exhibited greater corneal invasion length and area, thickness, and vascular density compared with quiescent pterygium. Increased thickness in the active stage likely results from stromal fibrovascular proliferation. Greater vascular density may reflect increased angiogenesis. Shorter vessel length in active pterygium may be attributable to immature neovascularization. Furthermore, GO enrichment analysis showed that upregulated DEPs were associated with sprouting angiogenesis. Similar to our study, a study using histopathology and IHC showed that vascular density was significantly greater in active than in quiescent pterygium.^26^ In the clinic, the degree of pterygium activity and congestion is a subjective judgment of the clinician and cannot be accurately quantified. Previous studies have shown that vessel density measured using AS-OCTA can effectively differentiate pterygium from normal conjunctiva.^10^ Our study further showed that there is still a statistical difference in vessel density between quiescent and active pterygium. Also, the parameters measured by AS-OCTA were the most associated with the DEPs. Thus, AS-OCTA can be used to assist clinicians in staging pterygium to select appropriate treatment options.

To investigate the differences between the two stages, we conducted a proteomics study. Proteomic profiling revealed that immune-related alterations were a consistent finding across complementary analytical approaches, including GO, KEGG analysis, the PPI network, and GSEA. All three top enriched KEGG pathways were immune associated and functionally interconnected: complement components can directly activate neutrophils and promote NET formation; upon activation, platelets release factors such as THBS1, which can modulate immune cell functions and further stimulate NET generation^27^^,^^28^; in turn, NETs provide a structural scaffold that facilitates platelet and immune cell interactions, thereby amplifying inflammatory and immune responses.^29^^,^^30^ Consistent with this theme, all five upregulated DEPs included in the PPI network were linked to the complement system.

Among the upregulated proteins, THBS1 was prioritized for multilevel validation based on a convergence of bioinformatic, statistical, and biological criteria. First, it functioned as a relatively central hub within the PPI network, suggesting a key regulatory role. Second, its expression showed significant correlations with clinically relevant parameters of disease activity. Third, its biological functions align with the pathophysiological processes underlying pterygium progression.^31^^,^^32^ THBS1 is a matricellular glycoprotein known to regulate cell adhesion, migration, and immune responses.^33^ Critically, it is involved in angiogenesis, where it may exert a dual role,^34^^–^^36^ and is a major physiological activator of transforming growth factor‑β1, a central mediator of fibrosis.^37^^–^^39^ Thus, its functional profile is uniquely pertinent to the fibrovascular remodeling that characterizes disease advancement.

Although ELANE and fibrinogen chains (FGA and FGB) demonstrated comparable or slightly higher AUC values in ROC analyses, ROC performance was not used as the sole selection criterion. In our cohort, the fibrinogen chains did not show significant correlations with clinical parameters, and ELANE lacked significant associations with structural or hemodynamic features. Therefore, THBS1 was prioritized based on the integration of network centrality, clinical correlation, and biological relevance.

Therefore, we further verified THBS1 using ELISA, IHC, and RT‑qPCR. The results showed that THBS1 was elevated in active-stage pterygium compared with quiescent-stage pterygium. In addition, AUC calculations suggested that measuring THBS1 levels may aid in distinguishing between eyes at the active and quiescent stages of pterygium. Meanwhile, studies have indicated that THBS1 is a possible genetic marker of pterygium formation.^36^ Notably, prior studies have reported lower THBS1 levels in pooled pterygium tissue vs. normal conjunctiva, with THBS1 undetectable in a substantial proportion (44.2%) of cases, highlighting significant interindividual heterogeneity.^40^ Our study supplements this by suggesting that THBS1 levels may exhibit dynamic changes across different developmental stages of pterygium.

To minimize confounding from site-specific biological variation and enhance the reliability of our findings, all pterygium tissues analyzed in this study were derived from the nasal region, the most common clinical type.^41^ This nasal predilection is primarily attributed to the greater susceptibility of the nasal conjunctiva to ultraviolet focusing, tear flow-mediated accumulation of pathogenic factors, and local mechanical irritation.^42^^–^^44^ In contrast, temporal pterygium, although less common, may involve different etiological mechanisms and warrants independent investigation. Therefore, our findings are specific to nasal pterygium, and extrapolation to temporal pterygium should be made with caution.

This study has several limitations. First, the clinical staging of pterygium, although standardized, retains an element of subjectivity. Second, the study was designed as an exploratory, discovery-phase proteomic analysis with a limited sample size. This design prioritizes hypothesis generation but inherently limits the statistical power for rigorous multiple-testing correction at the single-protein level, which is why we used GSEA to identify coordinated pathway-level signals. Consequently, the proteomic findings, particularly the role of individual candidates like THBS1, require validation in larger, independent cohorts. Third, the validation of THBS1 by ELISA was also performed in a small sample set, further underscoring the need for expanded confirmation. Finally, the absence of a healthy conjunctival control group limits the interpretation of disease specificity for the identified protein changes. Our findings, therefore, reflect stage-associated differences within pterygium rather than alterations relative to normal tissue. Future studies incorporating healthy controls are needed to fully contextualize these results. Moreover, as a discovery-phase proteomic study, causal relationships require further validation in future mechanistic investigations.

Herein, integrated clinical and proteomic analysis of different pterygium stages underscores the importance of immune dysregulation and neovascularization in disease progression. Targeting these pathways may help to promote conversion from active to quiescent stages and reduce recurrence risk. Notably, THBS1 may serve as a potential biomarker, warranting further investigation into its functional role and clinical utility.

## Supplementary Material

Supplement 1
