# Supplementary material for: Proteomic and Clinical Characterization of Active vs. Quiescent Pterygium With Elevated Thrombospondin-1
Source: Transl Vis Sci Technol. 2026 Jun 24;15(6):30. doi: 10.1167/tvst.15.6.30 (PMC13313041; doi:10.1167/tvst.15.6.30)
Supplement: Supplement 1 [file tvst-15-6-30_s001.docx]

Supplementary Table 1. Upregulated Proteins in Advanced-Stage Pterygium

| UniProt | Protein name | Gene name | Fold Change | *P*-value |
| --- | --- | --- | --- | --- |
| P08246 | Neutrophil elastase | ELANE | 1.668 | 0.013 |
| P07996 | Thrombospondin-1 | THBS1 | 1.556 | 0.015 |
| P02671 | Fibrinogen alpha chain | FGA | 1.856 | 0.021 |
| P02679 | Fibrinogen gamma chain | FGG | 1.731 | 0.026 |
| P02675 | Fibrinogen beta chain | FGB | 1.876 | 0.030 |
| P49821 | NADH dehydrogenase [ubiquinone] flavoprotein 1, mitochondrial | NDUFV1 | 1.987 | 0.036 |
| P10155 | 60 kDa SS-A/Ro ribonucleoprotein | RO60 | 1.681 | 0.036 |

Supplementary Table 2. Downregulated Proteins in Advanced-Stage Pterygium

| UniProt | Protein name | Gene name | Fold Change | *P*-value |
| --- | --- | --- | --- | --- |
| O95359 | Transforming acidic coiled-coil-containing protein 2 | TACC2 | 0.664 | 0.039 |
| P62191 | 26S proteasome regulatory subunit 4 | PSMC1 | 0.649 | 0.047 |
| O43681 | ATPase GET3 | GET3 | 0.615 | 0.038 |
| Q16401 | 26S proteasome non-ATPase regulatory subunit 5 | PSMD5 | 0.592 | 0.018 |
| Q15008 | 26S proteasome non-ATPase regulatory subunit 6 | PSMD6 | 0.584 | 0.043 |
| Q15435 | Protein phosphatase 1 regulatory subunit 7 | PPP1R7 | 0.580 | 0.024 |
| Q99426 | Tubulin-folding cofactor B | TBCB | 0.536 | 0.046 |
| O60684 | Importin subunit alpha-7 | KPNA6 | 0.518 | 0.014 |
| P43686 | 26S proteasome regulatory subunit 6B | PSMC4 | 0.513 | 0.029 |
| Q53H12 | Acylglycerol kinase, mitochondrial | AGK | 0.484 | 0.018 |
| P20290 | Transcription factor BTF3 | BTF3 | 0.467 | 0.042 |
| Q9BUL8 | Programmed cell death protein 10 | PDCD10 | 0.444 | 0.050 |
| Q92820 | Gamma-glutamyl hydrolase | GGH | 0.415 | 0.039 |
| P34896 | Serine hydroxymethyltransferase, cytosolic | SHMT1 | 0.324 | 0.008 |
| P29218 | Inositol monophosphatase 1 | IMPA1 | 0.148 | 0.025 |

| Database | Gene Set | NES | *P*-value | FDR(q-value) |
| --- | --- | --- | --- | --- |
| GOBP | Negative regulation of translational initiation | -1.69 | <0.001 | 0.179 |
| GOBP | Negative regulation of immune system process | 1.63 | <0.001 | 0.179 |
| GOBP | Positive regulation of cell adhesion | 1.56 | <0.001 | 0.179 |
| GOBP | Regulation of DNA-templated transcription elongation | -1.71 | <0.001 | 0.179 |
| GOBP | Positive regulation of DNA-templated transcription elongation | -1.68 | <0.001 | 0.179 |
| GOBP | External encapsulating structure organization | 1.65 | <0.001 | 0.179 |
| GOBP | Negative regulation of adaptive immune response | 1.84 | <0.001 | 0.179 |
| GOBP | Sulfur amino acid metabolic process | -1.69 | 0.001 | 0.179 |

Supplementary Table 3. Gene set enrichment statistics from GSEA

**Supplementary Table 4. ROC analysis of upregulated proteins and clinical parameters**

| Variable | AUC | 95% CI | *P* value |
| --- | --- | --- | --- |
| **Proteins** |  |  |  |
| FGA | 0.771 | 0.592 – 0.951 | 0.003 |
| ELANE | 0.767 | 0.591 – 0.942 | 0.003 |
| THBS1 | 0.748 | 0.562 – 0.933 | 0.009 |
| FGB | 0.714 | 0.517 – 0.911 | 0.033 |
| NDUFV1 | 0.719 | 0.515 – 0.923 | 0.035 |
| RO60 | 0.690 | 0.489 – 0.892 | 0.064 |
| FGG | 0.700 | 0.493 – 0.907 | 0.058 |
| **Clinical Parameters** |  |  |  |
| Length of pterygium | 0.783 | 0.613 - 0.954 | 0.001 |
| Area of pterygium | 0.729 | 0.536 - 0.921 | 0.02 |
| Thickness of pterygium | 0.752 | 0.573 - 0.932 | 0.006 |
| Vessel density | 0.771 | 0.597 - 0.946 | 0.002 |
| Vessel length | 0.236  0.764 | 0.053 - 0.418  0.582 - 0.947 | 0.005 |

AUC, area under the receiver operating characteristic curve; CI, confidence interval.

Note on Vessel length: The AUC of 0.236 was calculated with active-stage pterygium as the positive event, which is consistent with the definition used for all other parameters in this table. If quiescent-stage is defined as the positive event, the corresponding AUC is 0.764. This indicates that shorter vessel length is associated with the active stage.
